# Diurnal variation of intraoral pH and temperature

**DOI:** 10.1038/bdjopen.2017.15

**Published:** 2017-06-30

**Authors:** Jung Eun Choi, Karl M Lyons, Jules A Kieser, Neil J Waddell

**Affiliations:** 1 Sir John Walsh Research Institute, Faculty of Dentistry, University of Otago, Dunedin, New Zealand

## Abstract

**Objective/Aims:**

The aim of this study was to measure continuously the intraoral pH and temperature of healthy individuals to investigate their diurnal variations.

**Materials and Methods:**

Seventeen participants (mean age, 31±9 years) wore a custom-made intraoral appliance fitted with a pH probe and thermocouple for two sets of 24 h, while carrying out normal daily activities including sleep. The continuous changes in intraoral pH and temperature were captured using a sensor placed on the palatal aspect of the upper central incisors. The collected data were categorised into different status (awake and sleep) and periods (morning, afternoon, evening and night). Both quantitative and qualitative analyses were conducted.

**Results:**

The intraoral pH change was found to show a distinctive daily rhythm, showing a 12-h interval between maximum (7.73) and minimum (6.6) pH values. The maximum and minimum values were found to repeat after 24 h. The mean pH over 48 h (two sets of 24 h) was found to be 7.27 (±0.74). There was significant difference found in pH when subjects were awake and asleep and different periods during the day (*P*<0.001). The mean intraoral temperature was 33.99 °C (±4.9), with less distinctive daily rhythm compared with pH. There was a significant difference found in temperature depending on the time of the day, except between morning and afternoon (*P*=0.78).

**Conclusion:**

Our results showed that there is a distinctive daily, circadian-like pattern in intraoral pH variation over a 24-h period, which has been considered as one of the risk factors in sleep-related dental diseases.

## Introduction

A key paper on circadian rhythms in saliva secretions was published by Dawes in 1972,^1^ which investigated the relationship over a 24-h period between saliva flow rate, oral temperature and the hours (times) of a 24-h day. He found that stimulated and unstimulated saliva flow and oral temperature all showed significant circadian rhythms. In a follow-up study, Dawes^2^ reported that secretion levels of salivary substances were also found to follow a circadian rhythm. Adequate salivary flow and saliva content were directly related to health status as it plays an important role for effective nutrition, maintenance of body functions and especially oral homoeostasis.^3–6^ Recent studies have reported that salivary glands, like other human organs such as the kidneys, contain a circadian clock.^3,4^ Zheng *et al.* ^3^ confirmed that the circadian clock of salivary glands is a peripheral clock, which regulates the type, amount and content of saliva as well as the fluid secretion.

Both medical and dental practitioners have encountered patients who have dental diseases that are postulated to have a link with circadian rhythms.^4^ There is also increasing evidence that intraoral acidity (salivary pH) has a close link with circadian rhythm and sleep, similar to salivary flow rate. Dental erosion, which is chemical-mediated wear of teeth due to low pH in the oral cavity, is commonly found in patients with dry mouth, gastroesophageal reflux disease and those with sleep-related issues.^7–11^ These groups of patients share the reduction in salivary flow rate as a common cause.

Lowered quantity and quality of saliva means there is less saliva to buffer a decrease in intraoral pH to provide adequate protection for teeth from acid attack.^12^ Moreover, the saliva secreted from different locations in the mouth (for example, the partotid, submandibular and sublingual salivary glands) varies in composition, which is found to influence the rate of dental erosion to various degrees.^13,14^ Since the patients with sleep-related disorders, such as sleep bruxism, sleep-related xerostomia and nocturnal gastroesophageal reflux disease, are even at a greater risk of developing dental diseases such as caries and dental erosion, a link between intraoral pH and circadian rhythm is presumed.^8,9^ It has also been reported that night-shift workers are at a higher risk of developing dental erosion.^12,15^ In particular, previous studies have revealed the abnormal expression of clock genes in the salivary glands of these patients, which may contribute to their reduced salivary flow.^3,4^

Despite the significant role of pH on dental diseases, it has been difficult to monitor the salivary variables continuously for extended hours, including sleep especially the pH. Previous studies have only managed to measure the pH over a short period of time, and for selected teeth only.^16–21^ There is even less information on pH changes during sleep. In the studies to date, researchers have collected pH data only a few times during experimental sleep periods and involved waking the participants several times to obtain saliva samples. This does not represent the true continuous variation of the pH since the subjects’ sleep cycles and circadian rhythms is likely to have been disrupted.^16–21^ There are various devices and techniques developed to measure intraoral pH long term with minimal interference during daily activities and sleep. However, due to the limited number of participants, the link between pH and circadian rhythms cannot be verified.^18–21^

Long-term measurement of intraoral temperature has previously been reported. Similar to pH-related studies, only a few investigations have involved continuous temperature measurement during function and sleep.^22–24^ Intraoral temperature is suggested to have a close relationship with pH. When the temperature is lowered, for example, by opening the mouth during sleep, the saliva will evaporate, leaving a reduced amount of saliva to buffer the acid. To the best of our knowledge, there are no studies that have investigated the relationship between circadian rhythms, and intraoral pH and temperature simultaneously over an extended period.

Our research group has developed and validated an intraoral appliance, which is able to measure the pH and temperature continuously for long term. In the authors’ preliminary study, a significant difference in pH and temperature was found between the participants depending on whether they were awake or asleep.^25,26^ Therefore, the purpose of the study was to measure intraoral pH and temperature in healthy individuals over a 24-h period to further investigate a possible relationship of intraoral pH and temperature with circadian rhythms. The data obtained in this study should serve as a basis for future studies on various sleep-related dental diseases.

## Materials and Methods

### Subjects

Ethical approval for the study was granted by the University of Otago (ref. H14/021). The subjects were given a questionnaire to evaluate their health status. The exclusion criteria were history of dental erosion, xerostomia, eating disorders, respiratory disorders, sleep disorders, allergy, intake of medication, mouth breathing, smoking, wearing of orthodontic appliances and restorations on upper anterior teeth. Only participants with a healthy oral environment (for example, no caries, erosion and hyposalivation) and with no relevant medical (no sleep and respiratory disorders) history were included. All participants received verbal and written information concerning the study and gave written consent to participate. A total of 17 healthy volunteers (12 females and 5 males) with a mean age of 31±9 years participated.

### Instrumentation

Participants were invited to the University of Otago Faculty of Dentistry clinic and an impression of their upper dental arch was taken with an irreversible hydrocolloid (Aroma Fine Plus, GC, Tokyo, Japan), which was then cast in vacuum-mixed dental stone (Hydrocal 105, USA Gypsum, Chicago, USA). A custom-made intraoral pH and temperature-measuring appliance was constructed for each participant as per Choi *et al.* ^25^ Briefly, the appliance consisted of a thin, flexible vacuum-pressed EVA Bio-bleach plastic (1.0 mm) housing the pH probe (ResTech, San Diego, CA, USA) and the thermocouple sensor (Lascar Electronics, Erie, PA, USA). The probe and sensor were each connected to its own data-capturing device. The pH and temperature-measuring sensors were placed behind the central incisors, where most dental erosion is reported.^16,17^ It is also reported to show the maximum variation of intraoral temperature, due to the airflow during inhalation.^27,28^ A new device was made with new probes after 1-week break to increase the accuracy of the result.

### Data recording

**Figure 1:**
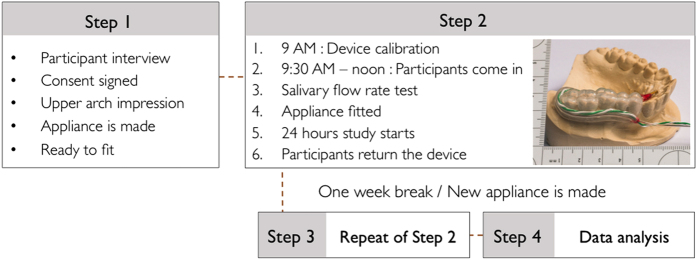
Study flow chart and intraoral appliance to measure the pH and temperature.

On the morning of the trial day (between 0900 and 1200 h), the device was calibrated according to the manufacturer’s instructions and the subject’s unstimulated salivary flow rate was measured using a 5-min spit technique.^29^ This was done to ensure that all participants were healthy saliva secretors. Following the salivary flow rate test, the appliance was fitted in the participant’s mouth. Each participant was asked to wear the device for a 24-h period and was encouraged to participate in normal daily activities. The participants were asked to record all daily activities in detail including their diets. Once the experiment started, each participant wore the appliance continuously except at meal times and in the shower. This was performed to avoid food residues getting into the probe or water getting into the data transmitter. The appliance was kept in distilled water at room temperature when not being worn, so that the pH probe would not dry out. During the 24-h period, pH was measured at 1 Hz (one per second), whereas temperature was measured once per 10 s (0.1 Hz). Although the devices were in place, eating and taking a shower were not allowed to avoid food residues masking the sensors and water getting into the electronics. The data collection was done using a hybrid sampling method (linked-cross sectional); 17 subjects participated in two study sets of 24 h, with a 1-week period between (Figure 1). During the 2 days of study, all participants were asked to fill out a detailed log of daily activities in detail, which enabled the investigators to track down when the participants had the device in and out of the mouth, time that the subjects went to sleep, nocturnal awakenings lasting more than 30 min and time the subjects woke up. The collected data were downloaded from the recorders and retrieved by software provided by the manufacturers (View Lite, ResTech, San Diego, CA, USA for pH and EasyLog USB, Lascar Electronics Erie, PA, USA for temperature measurement).

### Data analysis

**Figure 2:**
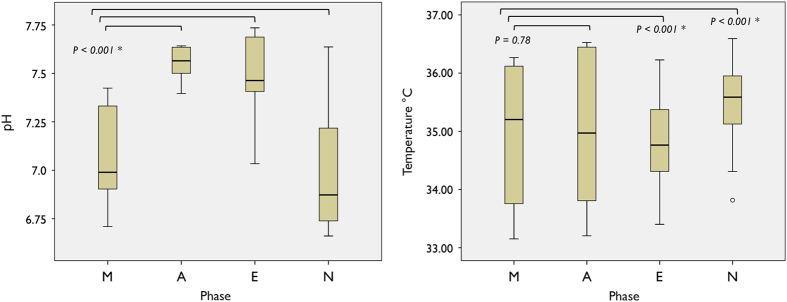
Median intraoral pH and temperature based on 48 h data collection, first and third quartile (box) and minimum and maximum (whiskers) for the different time periods categorised according to morning (M), afternoon (A), evening (E) and night (N) over a 24-h time period; significant differences between groups are indicated as *P* values (significance*P<0.05).

**Figure 3:**
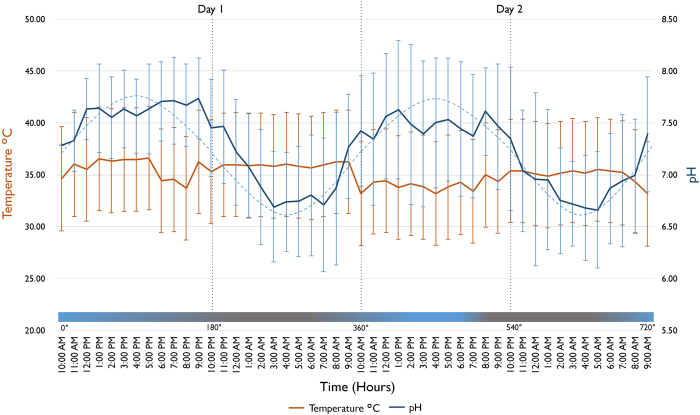
The total mean (with s.d.) of intraoral pH (top blue line, *y* axis to the right) and temperature (bottom orange line, *y* axis to the left) of all subjects over 48 h (time, *x* axis); dotted blue line represents the best fitting moving average trend of pH variations over 48 h showing a sinusoidal wave.

The recording portions corresponding to intervals when the appliance was not worn (for example, meal time and showering) were identified and removed. The study data were categorised into several measurement phases; awake or sleep and morning (M; 0600–1200 h), afternoon (A; 1200-1800 h), evening (E; 1800-0000 h), night (N; 0000-0600 h). The categorised data were summarised using descriptive statistics; mean, s.d., maximum and minimum (Tables 1 and 2 and Figure 2). Smooth lines and markers are fitted over the data to show the daily rhythm of intraoral pH over 48 h (Figure 3). Estimation of the variance was investigated as the total s.d. as well as a coefficient of variation (%). For comparison and correlation between the groups, non-parametric Wilcoxon-signed rank test and the Kruskal–Wallis test were used, respectively. Box and whisker plots with 95% confidence interval notches were used to determine the statistical differences between measurement phases. All statistical analyses were performed by SPSS version 22 for PC (SPSS, IBM, Armonk, NY, USA) and *P* value of <0.05 was considered statistically significant.

## Results

The average salivary flow rate of the 17 participants was 0.98±0.42 ml/min, confirming that they were healthy saliva secretors.^15^ All participants wore the pH and temperature-measuring intraoral device for up to 48 h over two periods of 24 h. It was found that the participants spent 8.1±1.95 h sleeping per day during the experiment.

The mean pH over 48 h (two sets of 24 h) was 7.27 (±0.74). When participants were awake, the intraoral pH varied depending on the time of the day; the difference in pH when the participants were awake compared with when they were asleep was found to be significant (*P*<0.001) and the results are presented in Tables 1 and 2 and Figure 2. The maximum pH was 7.73 and minimum pH was 6.6 (Table 2).

The mean intraoral pH and its variation during the 48 h are presented in Figure 3. The maximum pH values were found in the afternoon, between 1600 and 1900 h, whereas the minimum values were found between 0400–0700 h when the participants were sleep. It was found that there were 12-h intervals between maximum and minimum pH values and the pH returned to its maximum and minimum values after 24 h, showing a circadian-like rhythmic pattern (Figure 3).

Less distinctive and rhythmic patterns to the pH data were found in the changes of intraoral temperature. The mean temperature from the 48 h of study of all participants was 33.99 °C (±4.9), with fluctuations over the experimental periods. The Kruskal–Wallis analysis revealed that the differences in intraoral temperature were significant between all four measurement phases during the day except between morning and afternoon (*P*=0.78) as shown in Figure 3. However, there was significant difference found in intraoral temperature when the data were categorised into awake and sleep status (*P*<0.001).

## Discussion

Previous studies have focused on establishing a link between salivary variables and circadian rhythms since it reveals useful information on the need for specifying collection times, initiation of sleep-related dental diseases.^3,4^ The first papers on circadian variation in salivary variables were published by Dawes,^1,2^ who investigated salivary flow rates, the composition of saliva as well as intraoral temperature. There is increasing evidence supporting the link between resting salivary flow and intraoral pH. For example, it has been shown that a decrease in the salivary flow rate results in a decrease in intraoral pH due to reduced intraoral buffering capacity and acid clearance.^5,7,10,11^ However, it has not been possible or practical to track salivary pH and flow rates long term in locations especially where there is a risk of dental diseases such as dental erosion.

Since the salivary flow rate and salivary composition are difficult to track in real time, the collection of salivary pH has been attempted by many researchers as a way of monitoring the saliva flow rate. An early study by Ferguson and Fort,^16^ where submandibular pH was measured every few hours, described the existence of a circadian variation as the maximum and the minimum pH values were separated by a 12-h interval with smooth variation between the points. This trend was also confirmed in our current study. The maximum and minimum pH values were found to appear 12 h apart and return to the same value every 24 h; around 1600 h for the maximum pH and 0400 h for the minimum. The mean waveform of intraoral pH is found to be roughly sinusoidal and these patterns present presumptive evidence of a circadian rhythm (Figure 3 and Table 2). In fact, the finding of pH values peaking in the late afternoon and the low pH during sleep matches closely with that found by Dawes^2^ in the variations of salivary flow rate. His suggestion on the idealised effect of sleep on salivary flow rate is apparent in the trend found in the current study. This trend for a variation in pH in a 24-h period is linked to the findings of previous studies on circadian gene expression involving the salivary glands,^3,4^ since oral pH is governed by the quantity and the quality of saliva secreted from various glands.

The sleep-and-wake cycle is found to have a close relationship with changes in oral pH, that is, the pH starts to fall when individuals fall asleep and at the end of sleep period coincides with the rising phase of the pH curve (Figure 2). Although this pH pattern with a nocturnal decline during sleep is similar to that previously described by Watanabe *et al.*,^21^ our findings present a more detailed illustration of the pH variations during sleep. Moreover, the study by Watanabe *et al.* ^21^ involved the collection of saliva and measuring the values outside the mouth, which decreases the accuracy of the values. In addition, the night-time collection was also limited to three measurements, just prior to sleep, midnight and after waking. Therefore, it is possible that critical pH-related information may have been missed during the non-measured sleep hours.

Some research groups have developed intraoral appliances to track pH continuously from a few minutes to 24 h.^17–20,26,30^ However, these studies had a limited number of participants and it was difficult to draw any conclusions on the link between intraoral pH and circadian rhythms.^26,30^ Watanabe *et al.* ^19^ used a wireless telemetry device to track intraoral pH and concluded that pH values rose gradually until 1500 h, where a plateau was reached. Our study also observed a gradual increase in pH until 1500-1600 h but instead of a plateau, the pH gradually decreased over time, from 1500-1600 h and during sleep. The fact that the study by Watanabe *et al.* ^19^ used only one subject to validate the use of their device, and that the participant was elderly and edentulous, may explain the disagreement with the findings of the present investigation. The older population and edentulous patients are known to have a reduced salivary flow rate.^5,12,15^

The merit of the current investigation over previous studies is that the pH and temperature data was collected continuously from 17 participants over two 24-h periods. This ensured that sampling was continuous and reproducible over at least one cycle of the anticipated daily rhythm and allowed the measuring of normative data and inter-individual differences. Using a non-invasive and customised intraoral appliance had the added advantage that any sleep disturbances were avoided.

Intraoral temperature was also measured simultaneously with pH for 48 h. However, a distinctive daily rhythm was not evident. The intraoral temperature was found to fluctuate more during daytime but remain stable during sleep (Figure 3). This pattern was in agreement with previous studies.^23,26,30^ Although the intraoral temperature showed no prominent circadian-like daily rhythm, a significant difference was found between morning to evening and night-time periods, no differences were found between the morning and afternoon time periods (*P*=0.78). These results were in contrast to our own previous pilot study, which showed no significant differences in intraoral temperature over a 24-h day.^25,26^ This may be due to the smaller sample size in the pilot study. Different activities carried out by participants (for example, sports) as well as different drinks consumed during the day may also have contributed to these contrasting results.

The measurement of salivary variables such as flow rate and saliva composition over a long-term period is still problematic. As intraoral pH is linked closely to changes in salivary variables such as flow rate and intraoral acid clearance, the recognition of a circadian-like daily variation in intraoral pH is important. The use of intraoral pH values could be a convenient way to study other variables including human circadian rhythm in general. The use of circadian rhythm timings could also be used to determine optimal saliva sampling collection times in future studies on saliva composition.^1,2,31,32^

The limitation of the current study was that the measurement was taken from one place within the oral cavity. A recent study by Wang *et al.* ^31^ also observed a clear pattern of biorhythm in salivary flow rates, however, the flow rates of the lower and upper labial glands showed different patterns. Hence, future studies should be conducted with measurements taken from various locations in the mouth for extended period of time.

Smaller peaks were also found from the data sets, especially during sleep, suggesting the existence of other potential rhythmic components but these were beyond the scope of the current study. More sophisticated analysis of the peaks in intraoral pH and collection of data from patients with sleep-related diseases may provide valuable information to study the links between the increased prevalence of dental diseases in patients with sleep-related diseases.

In conclusion, the results support the existence of a daily rhythmic pattern in intraoral pH and a less distinctive pattern in intraoral temperature. The findings from the current study may provide a foundation on which large-scale studies be conducted to answer many more important questions in the field of salivary and sleep research.

## Conclusions

In conclusion, the results from this study highlighted that:

1. There is a circadian-like daily rhythmic pattern in intraoral pH variation over a day, which has been hypothesised to be linked to various sleep-related dental diseases.
2. There is less distinctive and rhythmic patterns in the changes of intraoral temperature in individuals conducting normal daily activities.
3. There is significant difference found in both intraoral pH and temperature when the data is categorised into awake and sleep status.

## Figures and Tables

**Table 1:** The mean intraoral pH and temperature when the participants were awake or sleep

	*pH*	*Temperature*
Awake	7.56±0.64	34.34±2.74
Sleep	6.85±0.66	35.98±1.07
*P* value	<0.005*	<0.005*

Significance of the pH and temperature between two statuses is indicated by *P* values (significance *P<0.05).

**Table 2:** Mean, s.d., maximum, minimum and coefficient of variation (s.d./mean×100) of pH and temperature measured by continuous collection over 48 h from all subjects

	*pH*				*Temperature* °*C*			
	*Mean (±s.d.)*	*CoV (%)*	*Max*	*Min*	*Mean (±s.d.)*	*CoV %*	*Max*	*Min*
Total (48 h)	7.27±0.74	10.18			33.99±4.90	14.42		
Morning (0600-1200 h)	7.20±0.72	10	7.42	6.71	32.70±5.71	17.46	36.27	33.15
Afternoon (1200-1800 h)	7.65±0.51	6.66	7.64	7.4	33.11±5.89	17.79	36.59	33.2
Evening (1800-0000 h)	7.59±0.67	8.83	7.73	7.03	34.55±3.36	9.72	36.23	33.4
Night (0000-0600 h)	6.94±0.70	10.08	7.08	6.66	34.42±4.75	13.8	36	34.89

The values are summarised by four time periods during a 24-h day.
